# Airborne Movement of Antibiotic Resistance Genes Between Livestock Stables and Farmers’ Homes

**DOI:** 10.3390/microorganisms14040855

**Published:** 2026-04-10

**Authors:** Hesham Amin, Tina Šantl-Temkiv, Kai Finster, Vivi Schlünssen, Torben Sigsgaard, Inge M. Wouters, Martin Tang Sørensen, Andrei Malinovschi, Hulda Thorarinsdottir, Randi J. Bertelsen

**Affiliations:** 1 Department of Clinical Science, University of Bergen, 5021 Bergen, Norway; randi.j.bertelsen@uib.no; 2 Section for Microbiology, Department of Biology, Aarhus University, 8000 Aarhus, Denmark; 3 Department of Public Health, Institute of Environmental and Occupational Medicine, Aarhus University, 8000 Aarhus, Denmark; vs@ph.au.dk (V.S.); ts@ph.au.dk (T.S.); 4 Institute for Risk Assessment Sciences, Faculty of Veterinary Medicine, Utrecht University, 3508 Utrecht, The Netherlands; 5 Department of Animal and Veterinary Sciences, Aarhus University, 8830 Tjele, Denmark; 6 Department of Medical Sciences, Clinical Physiology, Uppsala University, 75185 Uppsala, Sweden; 7 Department of Anesthesia and Intensive Care, Landspitali University Hospital, 105 Reykjavik, Iceland; huldaros@landspitali.is

**Keywords:** airborne ARGs, livestock AMR transmission, environmental resistome, metagenomics livestock dust, One Health

## Abstract

Antibiotic resistance genes (ARGs) are prevalent in livestock environments due to antimicrobial use, yet their airborne dispersal into human-occupied indoor spaces remains poorly characterized. We investigated whether airborne ARGs disperse from livestock stables into farmers’ homes and surrounding outdoor environments. Electrostatic dust collectors were deployed in paired pig and cow stables and their associated homes in Jutland, Denmark, to collect settled airborne dust. Pooled samples were analyzed using shotgun metagenomic sequencing. ARG dispersal patterns were assessed using FEAST source tracking and ecological similarity metrics, including shared ARG ratios and Jaccard indices. Pig production systems exhibited higher antibiotic use and stronger resistome continuity with farmers’ homes than cow systems, reflected by greater FEAST contributions (P = 0.029) and Jaccard similarity (P = 0.029). Beta-diversity analysis supported higher compositional similarity between pig stables and homes (PERMANOVA R^2^ = 0.23, *p* = 0.052), whereas cow environments showed greater divergence (R^2^ = 0.41, P = 0.035). Across environments, tetracycline, macrolide–lincosamide–streptogramin B, and aminoglycoside resistance genes dominated, consistent with livestock-specific antibiotic use patterns. Supplementary indoor–outdoor comparisons across cow, pig, and chicken stables (from an independent 2024 sampling campaign not directly comparable to the 2008 EDC-based survey) revealed contrasting dispersal dynamics, with higher bacterial species spillover from cow stables but stronger ARG overlap from pig stables. Collectively, these findings are consistent with airborne ARG connectivity across occupational and environmental interfaces and support consideration of air as a potential pathway in One Health AMR surveillance.

## 1. Introduction

The transmission of antibiotic resistance genes (ARGs) between animal agriculture and human environments represents an important but insufficiently understood dimension of the antimicrobial resistance (AMR) crisis. Livestock production systems are well recognized as reservoirs of ARGs due to intensive antibiotic use [1,2]. However, far less is known about how these genes move through air and reach human-occupied indoor environments. Among such settings, farmers’ homes located near livestock facilities are subject to repeated dust and aerosol transfer via occupational activities and daily movement between stables and residential spaces [3]. 

Airborne particles generated within livestock stables contain complex mixtures of bacteria, extracellular DNA, and organic matter that can act as vectors for ARG dissemination [4]. Once aerosolized, these particles may infiltrate nearby homes through ventilation systems, open structural interfaces, or transport on clothing and hair [5]. Such exposure pathways are particularly plausible in small- to medium-scale farms where residential and animal production facilities share the same property and operate within short physical distances [6]. Consistent with this biological plausibility, a growing body of research has documented the presence and emission of ARGs in agricultural air environments, with field studies quantifying ARG concentrations and emission rates around dairy and swine facilities [7]. Critical reviews further identify agricultural operations as important atmospheric sources of resistance genes while emphasizing methodological heterogeneity and the need for improved source attribution and exposure assessment [8]. Despite this accumulating evidence, few studies have explicitly evaluated potential airborne transmission of ARGs between occupational livestock environments and adjacent residential indoor spaces using paired sampling designs.

To address this knowledge gap, we implemented a paired sampling design including cow and pig stables and the homes of farmers working within them in Jutland, Denmark. Settled airborne dust was collected using electrostatic dust collectors and analyzed using shotgun metagenomic sequencing with ARG annotation against the CARD database. This approach enables direct investigation of airborne-associated resistome connectivity using culture-independent, high-resolution metagenomic profiling. We applied source-tracking analysis (FEAST) alongside ecological similarity metrics, including shared ARG ratios and Jaccard indices, to quantify resistome overlap between occupational and residential environments. By combining passive airborne dust sampling with shotgun metagenomics, this study provides a novel framework for assessing environmental resistome connectivity across built environments. To further contextualize airborne ARG dispersal, we extended the analysis to include indoor and outdoor air samples from cow, pig, and chicken stables. This supplementary component enabled assessment of microbial and resistome exchange between built agricultural environments and surrounding outdoor air, providing ecological insight into the environmental mobility of airborne ARGs.

## 2. Materials and Methods

### 2.1. Antibiotic Consumption Data

Antibiotic consumption data for pigs and cattle in Denmark were obtained from national surveillance reports (DANMAP) for the year 2008 [9] representing country-level usage patterns. This period corresponds to the time frame during which airborne dust samples were collected from livestock stables and associated farmers’ homes. These reports provide detailed records of antimicrobial consumption by antibiotic class and livestock species. The objective was to contextualize resistome profiles in relation to species-specific antimicrobial use patterns.

To enable standardized comparisons across livestock types and antibiotic classes, usage data were expressed as Animal Daily Doses per 200 kg biomass (ADD200), a metric reflecting the number of treatment doses administered per standardized animal mass [10].

### 2.2. Livestock Stables and Associated Farmers’ Homes Samples

Indoor airborne dust was collected during 2008 from livestock stables and associated farmers’ homes in Jutland, Denmark, a region characterized by high livestock production density [11]. Dust was collected using electrostatic dust fall collectors (EDCs), a passive airborne dust sampling method consisting of a plastic holder equipped with an electrostatic cloth (exposure area: 0.0209 m^2^) to accumulate settled airborne particles. EDCs were deployed at approximately 1.5 m above floor level and exposed for 14 days, following previously validated protocols for indoor and agricultural environments [12]. Sampling was conducted throughout the year, and identical deployment durations were applied across all sampling locations. After exposure, EDC cloths were collected using sterile forceps, placed in sterile containers, and transported under controlled conditions. Samples were stored at −20 °C until DNA extraction. All samples were processed within a consistent time frame to minimize degradation and ensure comparability across sampling sites.

Dust from the EDC cloths and DNA extraction followed the protocol described by Vestergaard et al. [13]. DNA was extracted using the DNeasy PowerLyzer PowerSoil Kit (Qiagen, Hilden, Germany) with bead-beating performed in a TissueLyser (2 × 5 min at 50 s^−1^). Due to low DNA yields from individual EDC cloths, a pooling strategy was employed to obtain sufficient material for shotgun metagenomic sequencing. For each environment type (cow stables, cow farmers’ homes, pig stables, pig farmers’ homes), seven individual DNA extracts were combined to generate one pooled sample. This process was repeated four times per environment, resulting in four pooled samples per group and 16 pooled samples in total. The resulting DNA extracts were then subjected to library preparation, shotgun metagenomic sequencing, quality control, taxonomic profiling, and ARG identification as described below.

**Figure 1:**
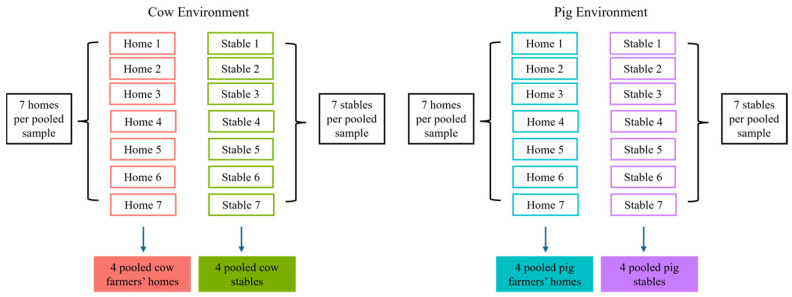
Overview of the paired sampling and pooling strategy across livestock environments. For each environment type (cow stables, cow farmer homes, pig stables, and pig farmer homes) seven individual EDC samples were combined into one pooled sample. This was repeated four times per group, resulting in four pooled samples per environment. Each pooled sample represents a matched group of samples collected from either homes or stables, allowing direct comparison between where farmers work and where they live. Arrows indicate the pooling process from individual samples into pooled samples for each environment.

Pooling was conducted within matched occupational–residential contexts, such that each farmer’s home pool corresponded to a pooled sample derived from the associated stable where the farmers worked (Figure 1).

To evaluate airborne microbial and ARG dispersal beyond indoor stable environments, supplementary indoor and outdoor air sampling was conducted in 2024 at livestock facilities located at the Department of Animal and Veterinary Sciences, Aarhus University (Viborg, Denmark). Indoor air was sampled using low-flow vacuum pumps (flow rate: 20 L/min) equipped with 0.22 µm polyethersulfone membrane filters, with two 24 h samples collected per livestock type (cow, pig, chicken). Outdoor air was collected approximately 30 m downwind of each stable using a high flow impinger system (Kärcher DS5800) (Alfred Kärcher GmbH & Co. KG, Winnenden, Germany), operated for 90 min at a flow rate of 3 × 10^3^ L/min, following the protocol described by Santl-Temkiv et al. [14]. Sampling locations were selected to capture representative airflow conditions relative to the stables. Negative controls were included for both indoor and outdoor sampling workflows. Collected filter and impinger samples were processed for DNA extraction and subjected to shotgun metagenomic sequencing following the same general workflow described below. These active air sampling approaches were applied to capture short-term airborne dynamics and were not designed for direct quantitative comparison with the passive EDC-based sampling used in the primary dataset, but rather to provide complementary evidence of environmental ARG dissemination.

### 2.3. Shotgun Metagenomic Sequencing and Resistome Profiling

The analytical workflow comprised sequential steps including library preparation, shotgun sequencing, quality control, taxonomic profiling, ARG identification, and downstream statistical analysis. Shotgun metagenomic sequencing was outsourced to Clinical Microbiomics (Copenhagen, Denmark). DNA libraries were prepared using the NEBNext Ultra Library Prep Kit for Illumina (New England Biolabs, Ipswich, MA, USA) following the manufacturer’s protocol. Sequencing was conducted on an Illumina NovaSeq 6000 platform (Illumina, San Diego, CA, USA), generating 150 bp paired-end reads. Raw sequencing reads underwent quality control, including adapter trimming and removal of low-quality reads, prior to downstream analysis. 

Reads were subsequently processed using the CosmosID bioinformatics platform (CosmosID, Germantown, MD, USA) for taxonomic profiling and ARG identification. CosmosID employs a high-resolution k-mer-based algorithm [15] for rapid and accurate classification of microbial sequences and ARG identification, leveraging curated reference databases including the Comprehensive Antibiotic Resistance Database (CARD, version 3.2.9) [16]. Relative abundances of ARGs were calculated based on the detection of ARG-specific k-mer signatures and normalized to reflect their proportional representation within each sample. Negative controls were included and analyzed alongside samples to assess potential background contamination and ensure data quality. 

### 2.4. Statistical Analysis

Statistical analyses were conducted in R (version 4.4.1). ARG relative abundances were aggregated at class and gene levels. Heatmaps were generated to visualize the top 20 most abundant ARGs and ARG classes. Alpha diversity was assessed using the Shannon diversity index. Differences across environment types were evaluated using Kruskal–Wallis tests followed by pairwise Wilcoxon rank-sum comparisons where appropriate.

Beta diversity was assessed using Bray–Curtis dissimilarity matrices and visualized via Principal Coordinates Analysis (PCoA). Group differences in resistome composition were tested using permutational multivariate analysis of variance (PERMANOVA) implemented via the adonis2() function. R^2^ values and associated *p*-values were reported.

### 2.5. Source Tracking and Transmission Metrics

To assess the potential movement of ARGs between environments, we applied source-tracking and ecological similarity analyses. FEAST (Fast Expectation–mAximization microbial Source Tracking) (version 0.1.0) was used to estimate the proportional contribution of livestock stable resistomes to the corresponding farmers’ home resistomes. In this framework, each stable sample was designated as the source and the paired home sample as the sink [17].

To complement source-tracking estimates, pairwise ecological similarity metrics were calculated. The shared ARG ratio was defined as the proportion of ARGs detected in the stable that were also present in the matched home. The Jaccard similarity index [18], was used to quantify compositional overlap, calculated as the number of shared ARGs divided by the total number of unique ARGs across both environments. Values approaching 1 indicate greater resistome similarity.

This analytical framework was extended to indoor–outdoor comparisons across livestock types. Pooled indoor stable samples were treated as sources and outdoor air samples as sinks. Analyses were conducted for both ARG profiles and species-level bacterial taxonomic composition.

## 3. Results

### 3.1. Antibiotic Consumption Patterns in Pig and Cow Farming

**Figure 2:**
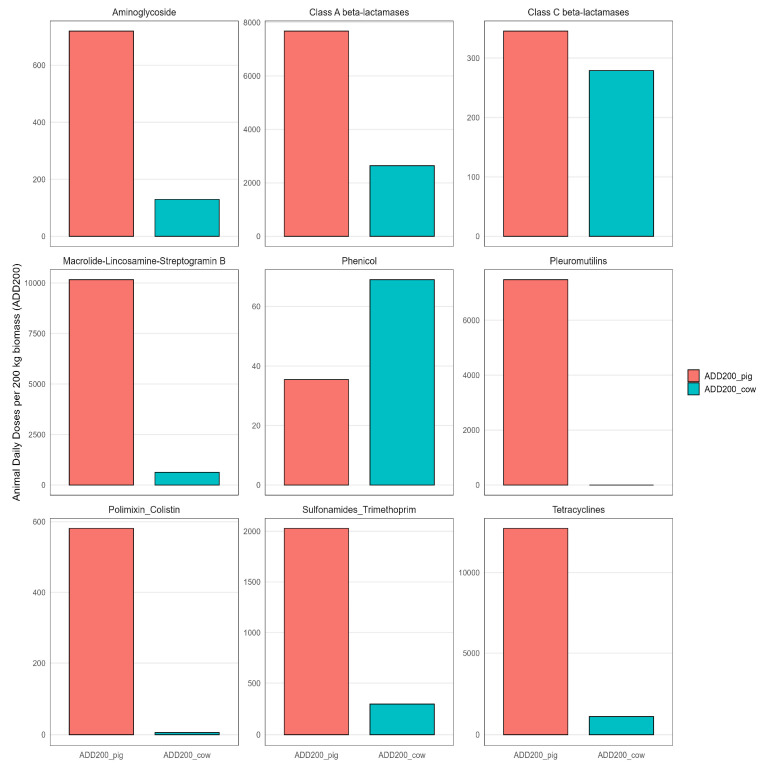
Pig production systems exhibit substantially higher antibiotic use across multiple classes compared to cow systems. Antibiotic consumption in Danish pig and cow farming in 2008. Bar plots show Animal Daily Doses 200 (ADD200) for pigs (red) and cows (blue) across nine antibiotic classes. ADD200 represents the number of standardized daily treatment doses per 200 kg animal biomass.

Antibiotic usage data from Danish pig and cow farming in 2008 (corresponding to the same period during which airborne samples were collected) were expressed as Animal Daily Doses per 200 kg biomass (ADD200) and revealed marked differences between livestock types. Overall antimicrobial consumption was substantially higher in pig production systems than in cattle farming. Pig production exhibited higher ADD200 values across all major antibiotic classes, except for phenicols, which were more frequently used in cattle (Figure 2).

### 3.2. ARG Diversity and Community Structure

**Figure 3:**
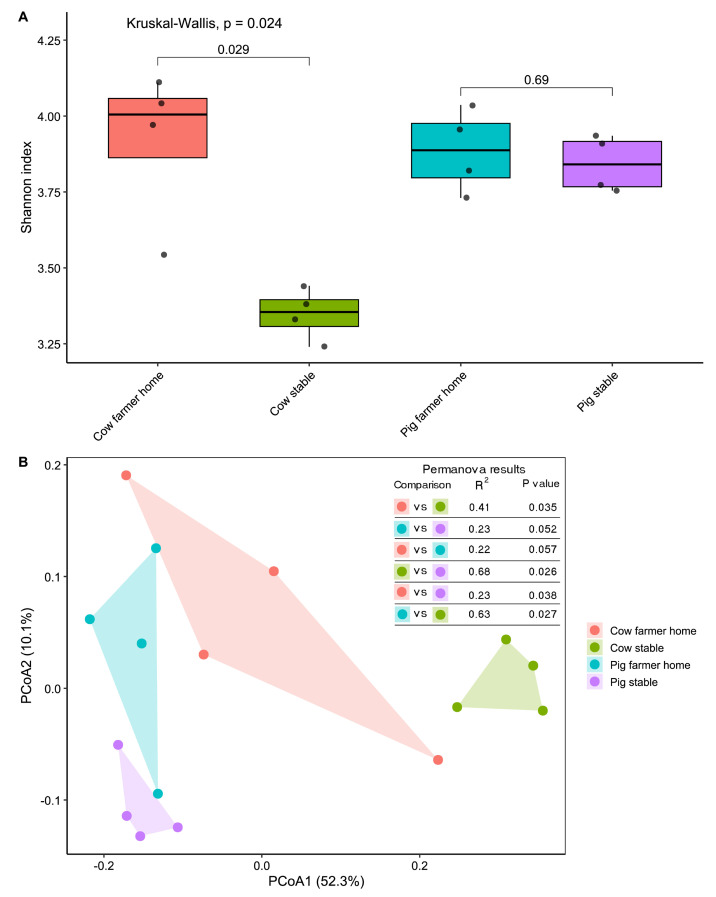
Pig-associated environments show greater resistome similarity between stables and farmers’ homes, whereas cow environments exhibit stronger differentiation. Alpha and beta diversity of resistome profiles across livestock and farmers’ home environments. (**A**) Boxplots showing Shannon diversity (alpha diversity) of ARGs across four sample types: cow stables, cow farmers’ homes, pig stables, and pig farmers’ homes. (**B**) Principal Coordinates Analysis (PCoA) based on Bray–Curtis dissimilarities showing clustering by environment type. Cow stable and home samples formed distinct clusters, whereas pig stable and home samples clustered more closely. PERMANOVA results are presented in the inset table. Colored points represent individual samples, and shaded areas indicate group clustering by environment type.

We assessed within-sample diversity (alpha diversity) of airborne ARGs across the four environment types using the Shannon diversity index (Figure 3A). A significant overall difference in ARG diversity was observed (Kruskal–Wallis *p* = 0.024). Pairwise comparisons indicated higher Shannon diversity in pig stables compared with cow stables. ARG diversity in cow farmers’ homes was significantly higher than in cow stables (Wilcoxon *p* = 0.029), while no significant difference was observed between pig stables and pig farmers’ homes (Wilcoxon *p* = 0.69).

To investigate differences in overall resistome composition across environments, we conducted a beta diversity analysis using Bray–Curtis dissimilarities and visualized the results through Principal Coordinates Analysis (Figure 3B). The PCoA plot revealed distinct clustering by environment type. Samples from pig stables and pig farmers’ homes were positioned on the same side of the ordination plot, although they formed separate clusters. In contrast, cow stables and cow farmers’ homes exhibited greater spatial separation within the ordination space.

These patterns were statistically supported by PERMANOVA. Resistome composition differed significantly between cow stables and cow farmers’ homes (R^2^ = 0.41, *p* = 0.035). A comparable but weaker separation was observed between pig stables and pig farmers’ homes (R^2^ = 0.23, *p* = 0.052). Additional pairwise comparisons indicated that cow and pig stables exhibited the greatest compositional divergence among all groups (R^2^ = 0.68, *p* = 0.026), with samples from each environment positioned on opposite sides of the PCoA plot.

### 3.3. ARG Composition and Dominant Genes

**Figure 4:**
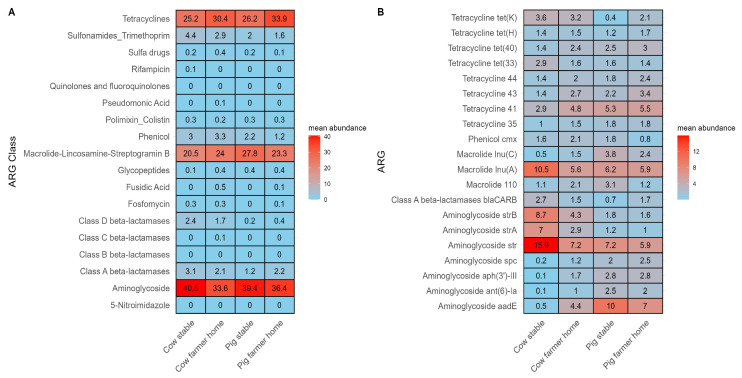
Tetracycline, macrolide–lincosamide–streptogramin B (MLS), and aminoglycoside resistance genes dominate airborne resistomes across both livestock and residential environments. Relative abundance of ARGs across livestock and farmers’ home environments. (**A**) Heatmap showing mean relative abundances of ARG classes across cow stables, cow farmers’ homes, pig stables, and pig farmers’ homes. (**B**) Heatmap showing mean relative abundances of the top 20 individual ARGs across the same environments. Color gradients represent scaled mean abundance values.

To characterize the composition of airborne resistomes across the four environments, we assessed mean relative abundances of antibiotic resistance gene (ARG) classes and individual ARGs (Figure 4). At the class level (Figure 4A), tetracyclines, macrolide–lincosamide–streptogramin B (MLS), and aminoglycosides consistently emerged as the dominant ARG classes across all environments. Together, these three classes accounted for up to 85% of the total ARG abundance within each environment.

At the individual gene level (Figure 4B), several tetracycline resistance genes, including *tet(K)* and *tet(H)*, were consistently detected across all environments. Aminoglycoside resistance genes were particularly prominent in cow stables, where *str* (15.9%), *strB* (8.7%), and *strA* (7%) were the most abundant. The macrolide resistance gene *lnu(A)* also showed notable enrichment, with peak abundance observed in cow stables (10.5%) and pig stables (6.2%). The full list of mean relative abundances for all ARGs across the four environments is provided in Supplementary Table S1.

### 3.4. ARG Transmission Between Stables and Farmers’ Homes

To assess potential transmission of ARGs from livestock stables to farmers’ homes, we quantified ARG similarity using three complementary metrics across eight matched pairs (four cow pairs and four pig pairs; Supplementary Table S2).

**Figure 5:**
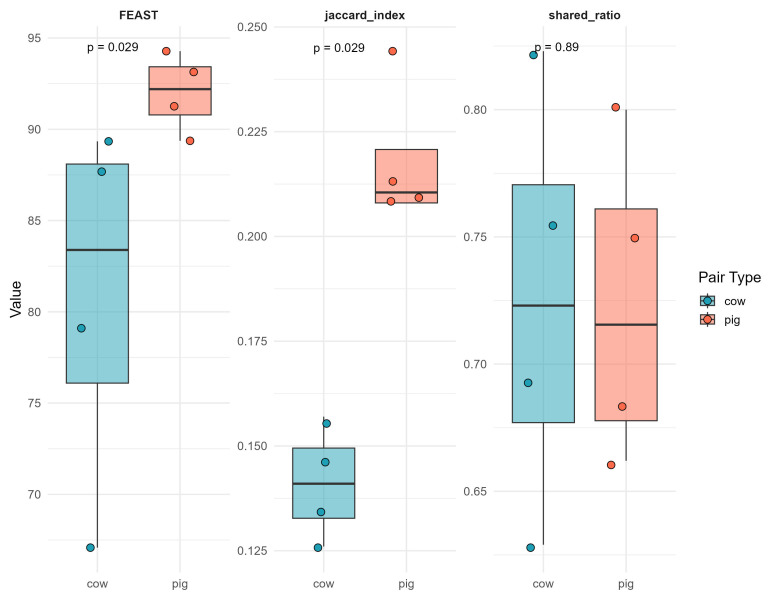
Pig environments exhibit stronger resistome similarity and source attribution between stables and farmers’ homes compared to cow systems, while shared ARG proportions remain comparable. ARG transmission between stables and farmers’ homes. Boxplots display pairwise comparisons of ARG overlap between livestock stables and farmers’ homes in cow and pig environments using three complementary metrics: FEAST source-tracking estimates (left), Jaccard similarity index (middle), and shared ARG ratio (right). Each dot represents one matched pair (*n* = 4 per group).

The FEAST source-tracking analysis estimated a significantly higher mean contribution of pig stables to pig farmers’ homes compared to cow systems (median: 92.2% vs. 83.3%; *p* = 0.029; Figure 5, left panel). Similarly, the Jaccard similarity index was significantly greater for pig pairs than cow pairs (median: 0.21 vs. 0.14; *p* = 0.029; Figure 5, middle panel), indicating greater resistome overlap in pig environments.

In contrast, shared ARG ratios (defined as the proportion of stable ARGs detected in the matched home) did not differ significantly between cow and pig systems (*p* = 0.89; Figure 5, right panel), indicating that the proportion of directly overlapping genes was comparable across livestock types despite differences in overall resistome similarity.

### 3.5. Dispersal of Airborne Bacteria and ARGs to Outdoor Environments

The following analyses are based on an independent 2024 sampling campaign, which serves as a supplementary (pilot) dataset and is not directly comparable to the 2008 EDC-based dataset used for the primary analyses. To evaluate the potential dispersal of indoor airborne microbes and ARGs into the outdoor environment, we compared indoor air samples from cow, pig, as well as chicken stables with chicken samples included as part of the 2024 indoor–outdoor sampling campaign to provide additional context on airborne dispersal across livestock systems. These indoor samples were compared to outdoor air samples collected approximately 30 m downwind. Source-tracking analysis (FEAST) and pairwise ecological similarity metrics (shared ratio and Jaccard index) were calculated for both bacterial species and ARGs.

**Figure 6:**
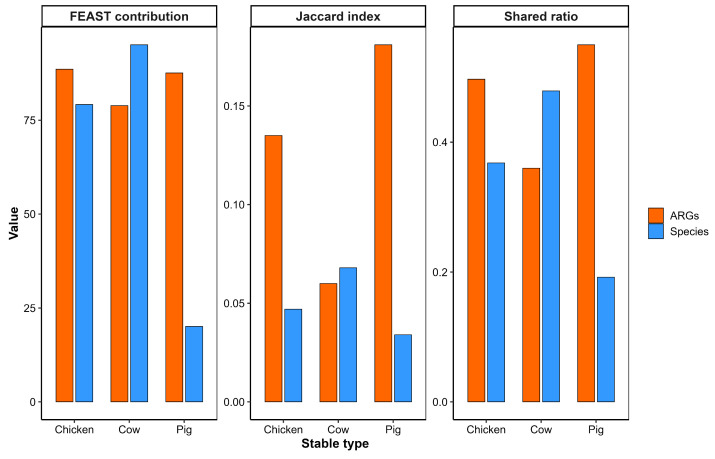
Cow stables show higher bacterial species dispersal to outdoor air, whereas pig stables exhibit stronger ARG transmission. Comparison of bacterial species and ARG transmission from indoor to outdoor air across cow, pig, and chicken stables. Bar plots display FEAST-estimated source contributions (%), Jaccard indices, and shared ratios for bacterial species (blue) and ARGs (orange). Data are derived from paired indoor and outdoor samples per stable type. Data are derived from an independent 2024 sampling campaign and are not directly comparable to the 2008 EDC-based dataset.

At the species level, cow stables exhibited the highest degree of microbial transmission to outdoor air, with 161 species shared (shared ratio = 0.479, Jaccard index = 0.068) and the highest FEAST contribution estimate (95.1%). Chicken stables showed moderate bacterial dispersal (shared ratio = 0.368, FEAST = 79.2%), whereas pig stables exhibited the lowest indoor–outdoor microbial overlap (shared ratio = 0.192, FEAST = 20.1%) (Blue bars in Figure 6) (See Supplementary Table S3 for all values).

In contrast, the pattern was reversed for ARGs. Pig stables exhibited the highest ARG transmission estimates to the outdoor air, with 99 ARGs shared (shared ratio = 0.55, Jaccard index = 0.181), and a FEAST estimate of 87.6%. Chicken stables also showed substantial ARG transmission (shared ratio = 0.497, FEAST = 88.6%). Cow stables, despite having the highest microbial spillover, showed the lowest ARG overlap with outdoor air, with a shared ratio of only 0.36 and a FEAST estimate of 78.9% (Orange bars in Figure 6) (See Supplementary Table S4 for all values).

## 4. Discussion

This study provides evidence for environmental resistome connectivity between livestock stables, farmers’ homes, and the surrounding outdoor environment, with airborne transmission representing a plausible but not exclusive mechanism Using a paired sampling design and shotgun metagenomic sequencing, we characterized airborne resistome profiles in pig and cow farming systems and evaluated transmission patterns using source-tracking and ecological similarity metrics. By integrating these findings with antibiotic usage data, we identified livestock-specific patterns in ARG burden and composition. Collectively, our results highlight the role of livestock type and antimicrobial use practices in shaping the dispersal and environmental distribution of airborne ARGs within an occupational–residential exposure framework.

### 4.1. Antibiotic Use and Resistome Structure in Livestock Environments

The composition and diversity of airborne ARGs in livestock environments were consistent with patterns of antibiotic usage and species-specific management practices. Danish surveillance data [9] indicated substantially higher antibiotic consumption in pig farming compared to cattle production, particularly for tetracyclines, macrolides, and aminoglycosides. These usage trends were aligned with the relative resistome composition of airborne dust, in which tetracycline and MLS resistance genes constituted a major fraction of detected ARGs.

At the gene level, tetracycline resistance genes such as *tet(K)* and *tet(H)* were consistently dominant in pig-associated environments, whereas aminoglycoside resistance genes including *strA* and *strB* were more prominent in cow stables. These compositional patterns are consistent with the therapeutic applications of these antimicrobial classes in each livestock type, for example, tetracyclines are widely used in swine production for growth promotion and disease prevention, whereas aminoglycosides are more commonly administered in cattle for the treatment of mastitis or respiratory infections. However, it is important to emphasize that resistome profiles were based on relative abundance rather than absolute concentration. A higher proportional representation of a given ARG class does not necessarily indicate a greater environmental load, but rather its dominance within the local resistome structure.

Beyond compositional profiles, analyses of alpha and beta diversity revealed additional distinctions in resistome structure between cow and pig systems. Cow farmers’ homes exhibited significantly higher Shannon diversity of ARGs compared to cow stables, which may reflect contributions from multiple ARG sources beyond occupational exposure alone. These could include human-associated microbiota, household materials, or community environmental inputs, as reported in previous studies of household dust [19,20]. In contrast, pig stables and pig farmers’ homes displayed comparable alpha diversity, indicating a more homogeneous resistome structure across occupational and residential environments. This resistome diversity pattern is consistent with previously reported taxonomic diversity trends observed across the same environments [11].

PCoA further supported these ecological patterns. Pig stables and pig farmers’ homes clustered more closely, indicating greater compositional similarity between these environments, whereas cow stables and homes exhibited clearer separation. These patterns were statistically supported by PERMANOVA, which showed stronger resistome divergence between cow stables and homes than between pig-associated environments. Notably, the greatest compositional divergence was observed between cow and pig stables themselves, highlighting the livestock-specific structuring of airborne resistomes and the ecological influence of animal production systems.

### 4.2. Stable-to-Home Transmission of ARGs

Our source-tracking analysis using FEAST and pairwise ecological similarity metrics yielded findings consistent with airborne movement of ARGs from livestock stables into farmers’ homes. Across all three-transmission metrics, stronger transmission signals were observed in pig-associated environments than in cow systems. Specifically, pig stables contributed a higher proportion of their resistome to the corresponding farmers’ homes, and Jaccard similarity indices indicated greater ARG compositional overlap between pig stables and homes, reflecting closer alignment in gene profiles. Collectively, these findings suggest that while both livestock systems experience stable-to-home ARG connectivity, pig environments may exhibit greater occupational–residential resistome continuity.

Several ecological and structural factors may contribute to this pattern. Higher antibiotic usage in pig farming systems may shape resistome composition, potentially enriching airborne ARG reservoirs and increasing the likelihood of downstream environmental dispersal [21]. Differences in daily farm practices may also influence exposure dynamics. Pig farming often involves frequent and close interaction between workers and animals, including feeding, cleaning, and handling activities that generate aerosolized dust particles [22]. While similar interactions also occur in dairy farming (e.g., during milking), their frequency and intensity may differ, potentially contributing to variation in aerosol generation.

However, structural characteristics of livestock housing should also be considered when interpreting these patterns. Pig production systems are typically more enclosed with controlled ventilation, whereas cow barns are often more open and naturally ventilated. In principle, such openness could facilitate greater environmental exchange between stables and surrounding spaces. The observed resistome patterns therefore likely reflect a combination of factors, including antimicrobial usage intensity, animal density, aerosol generation, and ventilation structure, rather than a single directional transfer mechanism. Airborne ARGs may be transported via dust particles, microbial carriers, or extracellular DNA [23], contributing to occupational–residential resistome connectivity.

While the observed patterns are consistent with airborne dissemination and supported by detection of ARGs in outdoor air surrounding livestock facilities, alternative transfer pathways should also be considered. Mechanical transport of dust via clothing, footwear, or farm equipment could potentially contribute to ARG presence in farmers’ homes, although farmers commonly change clothing after stable activities. The present study was not designed to experimentally disentangle these pathways. In addition, distances between stables and associated farmers’ homes were not systematically recorded, as the study design focused on matched occupational–residential environments rather than distance-dependent dispersion gradients. In addition, shared regional microbial reservoirs and non-directional exchange processes may also contribute to the observed resistome overlap, and the cross-sectional design does not allow inference of directionality or temporal dynamics. Therefore, the observed resistome connectivity most plausibly reflects environmental exchange between occupational and residential environments, potentially involving both atmospheric and indirect mechanisms.

These findings are consistent with previous studies indicating elevated AMR exposure potential in pig farming environments [6], and provide additional evidence that airborne pathways may contribute to resistome connectivity across livestock occupational and residential environments within a One Health framework. 

### 4.3. Indoor to Outdoor ARG and Microbial Transmission

Indoor–outdoor air comparisons revealed contrasting dispersal patterns between bacterial communities and ARG profiles across livestock systems. Cow stables were associated with the highest degree of bacterial species overlap with outdoor air, whereas pig stables showed stronger ARG transmission signals despite comparatively lower microbial dispersal.

An explanation for this pattern may relate to differences in antimicrobial usage intensity and manure management practices between livestock systems. Higher antibiotic use in pig production systems may select for microbial communities with greater ARG density per cell, potentially resulting in elevated ARG burdens even when fewer bacteria are dispersed [24]. In addition, fecal accumulation within pig stables may contribute to the generation of ARG-containing dust particles that become aerosolized through animal activity and barn operations [25,26]. This effect may be further influenced by housing characteristics, as the sampled pig stables were enclosed with controlled ventilation, whereas the cow stables were more open and naturally ventilated. By contrast, cow stables often employ more structured manure handling systems with reduced indoor fecal accumulation, which may facilitate microbial dispersal while limiting the release of ARG-enriched particulate matter.

Collectively, these findings indicate that airborne resistome dispersal is not necessarily coupled with whole-cell microbial dispersion. Instead, resistome transport patterns appear to reflect a combination of antimicrobial use intensity, manure handling practices, aerosol generation, and environmental persistence dynamics. These observations are consistent with prior reports of elevated airborne ARG levels in proximity to pig farming operations [25,27] and highlight the value of integrating resistome and microbial community data when evaluating environmental AMR dissemination within agricultural ecosystems.

### 4.4. Study Limitations and Methodological Considerations

Several limitations should be acknowledged. First, the pooling of samples, while necessary due to low DNA yield, limits our ability to assess variability within environments or to infer exposure patterns at the individual home level. Second, the study was geographically restricted to farms in Jutland, Denmark, and the findings may not be fully generalizable to other agricultural regions, livestock systems, or climatic contexts. In addition, the supplementary indoor and outdoor air sampling conducted in 2024 represents a separate campaign and was not intended for direct quantitative comparison with the 2008 dataset used for the primary analyses. Although structural characteristics of livestock buildings were comparable, temporal differences in farm practices and environmental conditions may have influenced resistome composition.

Finally, while shotgun metagenomic sequencing enables comprehensive detection of resistance genes, it does not provide information on whether these genes are associated with viable bacteria, located on mobile genetic elements, or actively expressed under environmental conditions [28]. As such, the functional mobility and transfer potential of detected ARGs could not be directly evaluated. Future investigations may benefit from integrating metagenomics with complementary approaches such as transcriptomics, culture-based assays, and plasmid characterization to better resolve the viability and mobility of airborne resistomes.

### 4.5. Implications for One Health and Environmental AMR Control

This study has important implications for One Health strategies and the future of environmental AMR surveillance. Our findings are consistent with airborne movement of ARGs between livestock stables and farmers’ homes, highlighting residential spaces, particularly those located near animal production facilities as potential zones of AMR exposure. This observation challenges the traditional separation between occupational and domestic environments in AMR risk frameworks and supports the inclusion of household settings in farm-associated health and surveillance policies.

The stronger resistome continuity observed in pig production systems suggests the potential value of species-specific mitigation strategies. Practical measures such as optimized ventilation systems, physical separation between stables and living spaces, and targeted air filtration technologies may help reduce airborne ARG dispersal [29]. In addition to indoor transmission pathways, the detection of ARGs in outdoor air sampled downwind of stables indicates that environmental dissemination may extend beyond farm buildings. Agricultural practices such as the outdoor application of pig manure could represent an additional environmental source of ARGs similar to those detected within pig stables, potentially contributing to localized resistome enrichment in surrounding air and soil matrices.

More broadly, these findings emphasize the importance of recognizing air as a potentially significant, yet underrepresented, pathway in AMR dissemination. While clinical environments and aquatic systems such as wastewater have traditionally dominated AMR monitoring and policy frameworks [30], airborne resistome dispersal remains comparatively under-investigated. Evidence from environmental hotspot studies indicates that ARGs can disperse over measurable distances via atmospheric transport, supporting the relevance of airborne pathways in regional AMR ecology [31]. Importantly, environmental and meteorological factors such as temperature, relative humidity, and wind speed are known to influence aerosol generation, transport, and persistence of airborne microorganisms and ARGs [4]. Although such parameters were not systematically captured in the present study, their integration into future sampling designs will be essential for refining our understanding of airborne AMR dynamics and improving predictive risk assessment models. Within this context, our findings contribute to a growing body of work suggesting that One Health AMR surveillance frameworks may benefit from explicitly incorporating atmospheric transmission pathways into risk assessment and mitigation strategies. 

## 5. Conclusions

In summary, this study provides evidence of resistome connectivity between livestock stables and farmers’ homes, that is consistent with airborne movement of ARGs, particularly in pig production systems where greater occupational–residential continuity was observed compared to cow systems. Indoor–outdoor comparisons further revealed divergent dispersal patterns, with cow stables associated with higher bacterial export and pig stables associated with stronger ARG transmission signals. Collectively, these observations highlight the relevance of occupational, residential, and environmental exposure pathways in shaping airborne AMR dynamics within livestock production ecosystems. These findings support incorporating airborne sampling and targeted mitigation (e.g., ventilation optimization, dust control, and filtration strategies) into farm-associated AMR surveillance and risk management.

## Data Availability

Sequencing data generated for this study have been deposited in the European Nucleotide Archive (ENA) under the study accession number PRJEB95762.

## Supplementary Materials

The following supporting information can be downloaded at: https://www.mdpi.com/article/10.3390/microorganisms14040855/s1, Table S1: Mean relative abundance of ARGs across livestock and farmers’ home environments; Table S2: Pairwise ARG similarity metrics between matched stable—home pairs; Table S3: Indoor—outdoor transmission metrics for bacterial species; Table S4: Indoor—outdoor transmission metrics for bacterial ARGs.

## Abbreviations

The following abbreviations are used in this manuscript:

ADD200	Animal Daily Doses per 200 kg Biomass
AMR	Antimicrobial Resistance
ARGs	Antibiotic Resistance Genes
CARD	Comprehensive Antibiotic Resistance Database
EDCs	Electrostatic Dust Collectors
MLS	Macrolide–Lincosamide–Streptogramin B
PM	Particulate Matter
R^2^	Coefficient of Determination
PERMANOVA	Permutational Multivariate Analysis of Variance
PCoA	Principal Coordinates Analysis
